# Reproductive Health is pleased to announce a mandatory open data policy in the journal

**DOI:** 10.1186/s12978-016-0190-4

**Published:** 2016-06-13

**Authors:** Natasha Salaria, Amye Kenall, José M. Belizán

**Affiliations:** BioMed Central, London, UK; Institute for Clinical Effectiveness and Health Policy (IECS), London, UK

## Introduction

The field of global health is evolving and moving forward from not just securing open access publication to aid in the dissemination of research but further still, making the data underpinning the results of that publication open. Funders^1^ in the field, and recently the International Committee of Medical Journal Editors (ICMJE) [1], are increasingly requiring that researchers make data produced during their grants publicly accessible.

Although the push for more data sharing and transparency started with the genomics community, it is quickly spreading to the global health communities. Indeed, two major organisations in the field have already set up a strong infrastructure for data reuse and sharing. Many of us are already familiar with the power of open data through the WHO Global Health Observatory Data Repository [2] and the World Bank Open Data Catalogue [3], both of which are invaluable resources. In February 2016 the Wellcome Trust also joined ‘over 30 global health bodies in calling for all research data gathered during the Zika virus outbreak, and future public health emergencies, to be made available as rapidly and openly as possible’ [4]. This follows a consensus statement arising from a WHO consultation in September 2015 moving towards making this the global norm [5].

*Reproductive Health* wants to help spearhead this paradigm shift to ensure all data underlying the research published within the journal is publicly available to ensure transparency of research and contribute to enhancing research in the field.

## Why open data?

Open Data—such as that found in the WHO Global Health Observatory Data Repository and from the World Bank—is especially important in the field of global health. Public health on a global scale requires the statistical power of pools of data rather than individual datasets. We need a bird’s eye view to detect trends and combat global epidemics.

Examples of the benefits of sharing data to global health research exist beyond the community resources mentioned from the WHO and World Bank. The Consortium of Health-Orientated Research in Transitioning Societies (COHORTS) study is one such example [6]. Funded by the Wellcome Trust and led by Cesar Victora, one group of researchers pooled data from 5 major cohort studies on maternal and infant factors in low and middle income countries. This larger pool of data provided relevant information about the relevance of the first 1000 days of life on further quality of life and educational attainment and earnings. Pooling their data gave the researchers enhanced statistical power and enabled them to generate more information around the impact on the first 1000 days of life. This example highlights how greater data sharing through collaboration can greatly benefit the individual researcher, leading to more prestigious and more impactful publications.

But reuse of, and the potential for, open data is only one of the driving factors. Also key to this conversation around data access is transparency. Health interventions must be driven by evidence, and that evidence must be verifiable. Well established organisations in the global health community have overstated the case for certain health interventions, polarising views on the use of certain interventions. A good example is the secondary analyses of the WHO Antenatal Care trial performed by Vogel et al. [7]. The large, cluster randomized WHO Antenatal Care Trial concluded that a goal-orientated package of antenatal care with reduced visits seemed not to affect maternal and perinatal outcomes [8]. This secondary analysis of the WHO Antenatal Care Trial data indicates that there is an appreciable increased risk of fetal death at 32 to 36 weeks gestation for women receiving the goal-oriented, reduced frequency antenatal care package. In a Commentary regarding the publication of this secondary analysis, Hofmeyr and Hodnett [9] concluded that “this re-analysis was robust after adjustment for potential confounding factors, and that the increase in perinatal mortality (was) consistent with trends in the two other cluster randomized trials conducted in Zimbabwe [10], we find the evidence, that a reduced number of antenatal visits is associated with increased perinatal mortality, compelling” [9]. Derivatives of these conclusions are of great magnitude since the WHO Antenatal Care Trial paper and derived publications such as the WHO manual for the implementation of the new model (http://www.who.int/reproductivehealth/publications/maternal_perinatal_health/RHR_01_30/en/index.html) have impacted antenatal care practice in many low-income countries. What is needed in health interventions is an objective, depoliticized view of the evidence. That kind of clarity comes with data transparency. Increased data access will also help to catch mistakes in reported results, which inevitably happen.

## Ways forward

### Data policy

In line with the above and to solidify our commitment in ensuring research data is made publicly available, *Reproductive Health* will be introducing a mandatory data sharing policy. We will be recommending that authors publish their data before submission in the Dataverse repository [11], a general repository run out of Harvard which charges no fee for storing up to 5GB of data. We also recommend authors utilise the following checklist when planning to deposit their data into their chosen repository: http://mozillascience.github.io/checklist/ [12].

### Data notes

In addition to this policy change, *Reproductive Health* has introduced a ‘Data Note’ as an article type [13] which authors are now able to select during submission. This article type is 3–4 pages in length and makes datasets open ahead of research findings being published. We have also updated the Instructions for Authors to include a section on ‘Availability of supporting data’. If you have any queries regarding the introduction of this new policy, please feel free to contact the Journal Editorial Office on reproductivehealthjournal@biomedcentral.com.

We are excited about this new chapter in *Reproductive Health* and look forward to receiving your Data Notes.

## Footnotes

1 Such funders and international organisations include, for example, Médecins Sans Frontières/Doctors Without Borders, WHO, Bill and Melinda Gates Foundation, Wellcome Trust, UK Medical Research Council (MRC), US National Institutes of Health (NIH), US Centers for Disease Control and Prevention (CDC), and others.
